# Retraction: Gastroprotective Activity of Ethyl-4-[(3,5-di-tert-butyl-2-hydroxybenzylidene) Amino]benzoate against Ethanol-Induced Gastric Mucosal Ulcer in Rats

**DOI:** 10.1371/journal.pone.0294007

**Published:** 2023-11-10

**Authors:** The PLOS ONE Editors

Following the publication of this article [1], concerns were raised regarding reuse of results presented in Figs 6, 7, and 8. Specifically,

- The Fig 6d panel appears to partially overlap with Fig 9 panel G7 of [2, corrected in 3, retracted in 4] when rotated, despite being used to represent different experimental conditions.
- Fig 7a of this article [1] appears similar to Fig 8G of [5, retracted in 6], despite being used to represent different experimental conditions.
- Fig 7e of this article [1] appears to fully or partially overlap with the following results, despite being used to represent different experimental conditions:
  ○ Fig 7G of [5, retracted in 6]
  ○ Fig 4e of [7, corrected in 8]*
  ○ Fig 8C of [9]
  ○ Fig 4e of [10]
  ○ Fig 5F of [11]
  ○ Fig 5 panel G3 of [12, retracted in 13]
- Fig 8a panel of this article [1] appears to overlap with the following results, despite being used to represent different experimental conditions:
  ○ Fig 5a of [10]
  ○ Fig 6 panel G1 of [12, retracted in 13]
  ○ Fig 10A of [14]
  ○ Fig 6D of [15, retracted in 16]*

The authors did not provide a response to the concerns raised with this article and did not provide the data underlying this study for editorial review. Given the nature and extent of the issues, the *PLOS ONE* Editors are concerned about the reliability of data management and/or reporting for this study [1].

In light of the above concerns, the *PLOS ONE* Editors retract this article.

Some figure panels discussed above appear to report previously published material that are offered under a CC BY license, but the original articles were not attributed in [1]. For these images, the * by the citation, above, marks the oldest publication of the image of which PLOS is aware.

PH, AN, and MAA agreed with the retraction. MH responded but expressed neither agreement nor disagreement with the editorial decision. MFH, RMS, DAB, NSAW, and AA either did not respond directly or could not be reached. MAA stands by the article’s findings.

## References

[1] Halabi MF, Shakir RM, Bardi DA, Al-Wajeeh NS, Ablat A, Hassandarvish P, et al. (2014) Gastroprotective Activity of Ethyl-4-[(3,5-di-tert-butyl-2-hydroxybenzylidene) Amino]benzoate against Ethanol-Induced Gastric Mucosal Ulcer in Rats. PLoS ONE 9(5): e95908. doi: 10.1371/journal.pone.0095908 24800807 PMC4011731

[2] Saeed AL-Wajeeh N, Halabi MF, Hajrezaie M, M. Dhiyaaldeen S, Abdulaziz Bardi D, M. Salama S, et al. (2016) The Gastroprotective Effect of *Vitex pubescens* Leaf Extract against Ethanol-Provoked Gastric Mucosal Damage in Sprague-Dawley Rats. PLoS ONE 11(9): e0157431. doi: 10.1371/journal.pone.0157431 27689880 PMC5045201

[3] AL-Wajeeh NS, Halabi MF, Hajrezaie M, Dhiyaaldeen SM, Abdulaziz Bardi D, Salama SM, et al. (2017) Correction: The Gastroprotective Effect of *Vitex pubescens* Leaf Extract against Ethanol-Provoked Gastric Mucosal Damage in Sprague-Dawley Rats. PLoS ONE 12(5): e0179072. doi: 10.1371/journal.pone.0179072 28562654 PMC5451129

[4] The *PLOS ONE* Editors (2023) Retraction: The Gastroprotective Effect of *Vitex pubescens* Leaf Extract against Ethanol-Provoked Gastric Mucosal Damage in Sprague-Dawley Rats. PLoS ONE 18(11): e0294006. doi: 10.1371/journal.pone.0294006 PMC10637635 37948341

[5] Nordin N, Salama SM, Golbabapour S, Hajrezaie M, Hassandarvish P, Kamalidehghan B, et al. (2014) Anti-Ulcerogenic Effect of Methanolic Extracts from *Enicosanthellum pulchrum* (King) Heusden against Ethanol-Induced Acute Gastric Lesion in Animal Models. PLoS ONE 9(11): e111925. doi: 10.1371/journal.pone.0111925 25379712 PMC4224391

[6] The *PLOS ONE* Editors (2023) Retraction: Anti-Ulcerogenic Effect of Methanolic Extracts from *Enicosanthellum pulchrum* (King) Heusden against Ethanol-Induced Acute Gastric Lesion in Animal Models. PLoS ONE 18(11): e0294008. doi: 10.1371/journal.pone.0294008 PMC10637633 37948333

[7] Ismail IF, Golbabapour S, Hassandarvish P, Hajrezaie M, Majid NA, Kadir FA, et al. (2012) Gastroprotective Activity of *Polygonum chinense* Aqueous Leaf Extract on Ethanol-Induced Hemorrhagic Mucosal Lesions in Rats. Evidence-Based Complementary and Alternative Medicine, Volume 2012, Article ID 404012. doi: 10.1155/2012/404012 23365597 PMC3544547

[8] Ismail IF, Golbabapour S, Hassandarvish P, Hajrezaie M, Majid NA, Kadir FA, et al. (2018) Corrigendum to “Gastroprotective Activity of *Polygonum chinense* Aqueous Leaf Extract on Ethanol-Induced Hemorrhagic Mucosal Lesions in Rats”. Evidence-Based Complementary and Alternative Medicine, Volume 2018, Article ID 8961462. doi: 10.1155/2018/8961462 30647764 PMC6320006

[9] Ibrahim IAA, Hussein AI, Muter MS, Mohammad AT, Al-Medhtiy MA, Shareef SH, et al. (2022) Effect of nano silver on gastroprotective activity against ethanol-induced stomach ulcer in rats. Biomedicine & Pharmacotherapy 154. doi: 10.1016/j.biopha.2022.113550 35994814

[10] Al Batran R, Abdulla MA, Al-Obaidi MMJ, Hajrezaei M, Hassandarvish P, Fouad M, et al. (2013) Gastroprotective effects of *Corchorus olitorius* leaf extract against ethanol-induced gastric mucosal hemorrhagic lesions in rats. Journal of Gastroenterology and Hepatology, 28(8): 1321–1329. doi: 10.1111/jgh.12229 23611708 PMC3842111

[11] Sidahmed HMA, Azizan AHS, Mohan S, Abdulla MA, Abdelwahab SI, Taha MME, et al. (2013) Gastroprotective effect of desmosdumotin C isolated from *Mitrella kentii* against ethanol-induced gastric mucosal hemorrhage in rats: possible involvement of glutathione, heat-shock protein-70, sulfhydryl compounds, nitric oxide, and anti-*Helicobacter pylori* activity. BMC Complementary Medicine and Therapies. 13: 183. doi: 10.1186/1472-6882-13-183 38641576 PMC11027352

[12] Al Batran R, Al-Bayaty F, Jamil Al-Obaidi MM, Abdualkader AM, Hadi HA, Ali HM, et al. (2013) In Vivo Antioxidant and Antiulcer Activity of *Parkia speciosa* Ethanolic Leaf Extract against Ethanol-Induced Gastric Ulcer in Rats. PLoS ONE 8(5): e64751. doi: 10.1371/journal.pone.0064751 23724090 PMC3665813

[13] The *PLOS ONE* Editors (2023) Retraction: In Vivo Antioxidant and Antiulcer Activity of *Parkia speciosa* Ethanolic Leaf Extract against Ethanol-Induced Gastric Ulcer in Rats. PLoS ONE 18(11): e0294012. doi: 10.1371/journal.pone.0294012 PMC10637640 37948324

[14] Saremi K, Rad SK, Tayeby F, Abdulla MA, Karimian H & Majid NA (2019) Gastroprotective activity of a novel Schiff base derived dibromo substituted compound against ethanol-induced acute gastric lesions in rats. BMC Pharmacology and Toxicology 20: 13. doi: 10.1186/s40360-019-0292-z 30770761 PMC6377749

[15] Ketuly K A., Hadi AH A., Golbabapour S, Hajrezaie M, Hassandarvish P, Ali HM, et al. (2013) Acute Toxicity and Gastroprotection Studies with a Newly Synthesized Steroid. PLoS ONE 8(3): e59296. doi: 10.1371/journal.pone.0059296 23516624 PMC3596355

[16] The *PLOS ONE* Editors (2023) Retraction: Acute Toxicity and Gastroprotection Studies with a Newly Synthesized Steroid. PLoS ONE 18(11): e0294010. doi: 10.1371/journal.pone.0294010 PMC10637634 37948326

